# Embodied Imagination and Metaphor Use in Autism Spectrum Disorder

**DOI:** 10.3390/healthcare9020200

**Published:** 2021-02-13

**Authors:** Zuzanna Rucińska, Thomas Fondelli, Shaun Gallagher

**Affiliations:** 1 Centre for Philosophical Psychology, University of Antwerp, Rodestraat 14, 2000 Antwerp, Belgium; ZuzannaAleksandra.Rucinska@uantwerpen.be; 2 Interactie Academie, van Schoonbekestraat 33, 2018 Antwerp, Belgium; thomas.fondelli@telenet.be; 3 Department of Philosophy, University of Memphis, Memphis, TN 38152, USA

**Keywords:** autism spectrum disorder, imagination, metaphors, embodied cognition, enactivism, affordances

## Abstract

This paper discusses different frameworks for understanding imagination and metaphor in the context of research on the imaginative skills of children with autism spectrum disorder (ASD). In contrast to a standard linguistic framework, it advances an embodied and enactive account of imagination and metaphor. The paper describes a case study from a systemic therapeutic session with a child with ASD that makes use of metaphors. It concludes by outlining some theoretical insights into the imaginative skills of children with ASD that follow from taking the embodied-enactive perspective and proposes suggestions for interactive interventions to further enhance imaginative skills and metaphor understanding in children with ASD.

## 1. Introduction

In this paper we explore the notions of imagination and metaphor as they are predominantly used in discussions of Autism Spectrum Disorder (ASD). We begin by explaining that the concept of imagination referenced in such discussions is seen as strongly connected to (and even resting upon) linguistic capacities [1], just as the concept of metaphor is strongly connected to semantic skills [2]. In line with the findings that people on the autistic spectrum show linguistic deficiencies [3], many theorists have also suggested that the imaginative and metaphoric skills of autistic individuals are lacking. As described by Wing and Gould in 1979, children with ASD show abnormalities of symbolic and imaginative activities [4]. Imagination has been seen as a core difficulty in ASD, a thought that is shared in contemporary research as well [5,6]. Similarly, the use of metaphors (whether in conversation or in pretend play), is also reported to be diminished in ASD [7].

We do not think that this connection between language and imagination is a coincidence. The descriptions of individuals with ASD as having “malfunctioning” imaginative skills or showing “lack of” metaphoric thought are correlated to the linguistic conceptual frameworks that the researchers are working with [1,5], and to the methods they use to test for imaginative and metaphoric skills of people with ASD that their frameworks inspire. Those conceptual frameworks we will collectively name “linguistic frameworks”. The methods they inspire are training tools that focus on semantic manipulations. We will give examples of both below.

We propose a different understanding of imagination and metaphor, one understood through the lens of embodied and enactive accounts of cognition [8,9]. Embodied and enactive cognition (part of “4E Cognitive Science”—which understands cognition to be embodied, enacted, embedded and extended) thinks of cognitive processes as grounded in perceptual and sensorimotor systems and in explicit interaction with the environment. Embodied cognition refers to the idea that cognition involves both neural and extra-neural structures and processes, including, for example, morphological factors, peripheral and autonomic systems, and affect. Enactive cognition refers to the idea that some cognitive processes are adaptive and action-oriented, involving reciprocal interactions between the agent and its environment. Enactivism makes use of the ecological notion of affordances, which are relational possibilities for action emerging in engagements with objects and others in the world [10,11,12]. 

Our proposal involves a shift from the linguistic framework of imagination and metaphor, to the embodied and enactive framework. This allows us to re-examine the imaginative acts of individuals with ASD, including their engagement with metaphors, suggesting insights on the types of methods required to uncover their imaginative skills. Our proposal is that shifting the starting point from language to embodiment, and seeing imagination as an embodied and enactive process (together with seeing metaphors as affordances for interactions enabled by those embodied and enactive imaginings), opens up a new way of making sense of imaginative and metaphoric skills of people with ASD, including making sense of how to interpret their limitations. Moreover, shifting the conceptual starting point makes a difference in the methods that would count as appropriate for evaluating imaginative acts of people with ASD, and what interventions are deemed as necessary for developing their imaginative skills. We propose to focus on interactive methods where subjects interactively engage with metaphors (presented with the example of systemic dialogical therapy), rather than to focus only on linguistic trainings that exercise one’s semantic knowledge. The embodied and enactive perspective on imagination and metaphor use in ASD provides a needed revision to the current theory of imagination and its application to ASD and adds to the existing practical methods and interventions that aim to enhance imaginative skills of autistics. 

The paper develops in the following way. Section 2 will describe, in more detail, the linguistic framework in imagination and metaphor theory and how it has affected research on imagination of people with ASD. Section 3 will focus on explicating a different, explicitly embodied account of imagination; Section 4 will outline an enactive approach to metaphor use, as described by theorists of embodied and enactive cognitive science. Section 5 describes a case study from a practical therapeutic session with a child with ASD that makes use of metaphors, illustrating the embodied and enactive take on metaphors in practice. Section 6 concludes the paper with insights on metaphoric and imaginative skills of people with ASD that follow from taking the embodied-enactive perspective on imagination, followed by some suggestions for the types of interventions that our theory advocates.

## 2. The Linguistic Framework in Imagination and Metaphor Theory

In this section, we will first present some views on imagination and metaphor as connected to linguistic capacities. Wing and Gould identify imagination as a core difficulty in ASD [4]. In a continuous search to understand how people on the autism spectrum think differently, a new line of research has turned to the concept of voluntary and top-down imagination. Voluntary imagination refers to the act of imagining as done on purpose, whereby one creates a conscious sensory experience at will [13]. It is opposed to having involuntary sensory experiences, such as phantom perceptions that occur without concurrent or direct sensory stimulation, or imaginings triggered by an association. Voluntary imagination is said to enable complex semantic relationships. For instance, Vyshedskiy et al. [1] take voluntary imagination to be “responsible for mental juxtaposition of objects into novel combinations [without which] it is impossible to understand the difference between sentences with identical words and grammar” (p. 3). Similarly, top-down imagination refers to the mechanism of imagination involving the lateral prefrontal cortex (LPFC). Top-down imagination is also voluntary because it is “associated with human purposeful mental simulations” [5] (p. 103). It allows for prefrontal synthesis (PFS), understood as a “spatial combination of two or more objects from memory into a novel mental image” [5] (p. 91). PFS enabled by top-down imagination is “essential for many cognitive functions, including the understanding of flexible syntax [and] understanding of spatial prepositions” [5] (pp. 91–92). Vyshedskiy gives an example of how top-down imagination allows for understanding the difference between gramatically similar sentences: “Consider the two sentences: “A dog bit my friend” and “My friend bit a dog.” It is impossible to distinguish the difference in meaning using words or grammar alone, since both the words and grammatical structure are identical in these two sentences. Understanding the difference in meaning, and appreciating the misfortune of the 1st sentence and the humor of the 2nd sentence depends on the LPFC ability to flexibly synthesize novel mental images according to a presented description. Only after the LPFC forms these two different images in front of the mind’s eye, are we able to understand the difference between these two sentences” [5] (pp. 91–92). The suggestion is that voluntary/top-down imagination is needed for the development of linguistic proficiency. However, voluntary imagination is also taken to rely on the development of prior linguistic reasoning. The acquisition of voluntary imagination is taken to be the function of using recursive language in early childhood [1,14]. Thus, the relationship between imaginative skills and linguistic skills is seen as closely interlinked and mutually dependent. 

Moreover, several researchers have shown that people with autism have difficulties with metaphor comprehension [15,16,17]. Metaphor is “but one of many techniques, named and unnamed, for likening one thing to another by means of words” [18]. Metaphor is defined in linguistic terms as a figure of speech or literary device that involves using one term instead of another based on the conscious violation of established categories. Neisser [9] characterizes metaphor as a “puzzling phenomenon of language” (p. 29). He clarifies that some follow the Aristotelian substitution view of metaphor, where “a transfer of meaning is accomplished by substituting one word for another”, which places the metaphors in the ranks of “rhetorical devices and stylistic tropes” [9] (p. 29). To interpret a metaphor, on this view, “is simply to supply its non-metaphorical paraphrase” [9] (p. 30). Others follow the interaction view [19,20], which sees metaphors as requiring “a transfer of meaning in which one thing is explained by being changed into another thing or an emotion or idea” [9] (p. 29). The interaction view “shifts analysis from the metaphorical word to the entire metaphorical sentence on the grounds that metaphor requires a semantic context that forges the connection of one subject to another” [9] (p. 30). The interaction view, however, is still confined to language. Accordingly, Neisser criticizes the interaction view by arguing that it does not explain how the metaphorical vehicle selects its associations; and he modifies it to account for the modeling function of metaphors, proposing that “the modeling function of metaphor should be understood as an act of embodied imagination, i.e., as the generation of an imaginal model” (p. 32). We will return to Neisser’s position in later sections.

Within the linguistic framework, metaphor production is seen as an imaginative skill. Metaphors, such as calling fireworks “flowers in the sky”, in this framework, are said to require seeing similarities between two entities that are normally considered distinct and combining disparate objects into a novel mental image. Both metaphor comprehension and use are seen as grounded in symbolic and counterfactual thinking, one that requires specific language competences, such as semantic knowledge [2]. Interestingly, whereas people with ASD seem to do worse in the comprehension and production of conventional metaphors in comparison with their typical peers, several researchers found no differences between people with ASD and their neurotypically developing peers in the comprehension of novel metaphors [21,22,23], and even found that people with ASD did better than their neurotypical peers in production of novel metaphors [24,25]. Being able to see similarities between two distinct objects is characterized by Norbury as “a complex developmental process that requires the acquisition of a number of skills. In the first place, children must acquire sufficient world knowledge and have broad enough semantic representations to capture the comparison being made” (p. 384). To decide in what respects two objects are similar involves “analogical reasoning skills” (p. 385) that allow one to think through the mappings from source to target domain.

ASD is seen as involving challenges with imagination and metaphor comprehension understood through the linguistic framework, due to the delays individuals with ASD show with language acquisition. For example, top-down imagination is “known to be a common challenge” for children with Autism Spectrum Disorder [5] (p. 104), who are said to experience delays in the development of top down imagination and, more specifically, PFS. Vyshedskiy [5] draws a causal connection between this imaginative skill and linguistic skills, as seen in this passage:

*“The impaired PFS affects virtually every area of an individual’s verbal, cognitive and social functioning, including the lack of comprehension of flexible syntax and spatial prepositions (…) As a result, 30–40% of individuals diagnosed with ASD experience lifelong impairment of the ability to understand flexible syntax and spatial prepositions. (…) In fact, the PFS ability and the derivative capacity of understanding flexible syntax and spatial prepositions, may be the most salient differentiator between high-functioning and low-functioning ASD (p. 104).”*

According to Vyshedskiy [5], PFS disability goes beyond problems with interpreting syntax and grammar (even though people with ASD show difficulties in understanding the differences between sentences with identical words and grammar); it is a disability of “one of the mechanisms of top-down imagination” (p. 95). Imaginative capacities “malfunctioning” for children on the spectrum refer to poor mental integration and stimulus overselectivity. For instance, Dunn et al. [26] clarify that the ability to juxtapose or mentally integrate multiple cues “is known to be a common challenge for children on the spectrum” (p. 3), while Vyshedskiy et al. [1] clarify that the “autism community refers to the phenomenon whereby individuals cannot combine disparate objects into a novel mental image as stimulus overselectivity, tunnel vision, or the lack of multi-cue responsivity” (p. 3, see also [27]). Stimulus overselectivity refers to a phenomenon “whereby an individual focuses on only one aspect of an object or environment while ignoring others” [1]. Writing about children with ASD, Vyshedskiy et al. explain that: “When asked to pick up a red crayon under the table, a child with ASD may hyper-attend to the cue “crayon” and ignore both its location and the fact that it should also be red, therefore picking up any available crayon. It is often said that individuals with ASD “can’t see the forest for the trees”: they pay too much attention to specific parts, get lost in the details and miss the whole picture (or Gestalt)” [1] (p. 3).

Children with ASD are also seen as impaired in their metaphoric engagements because of the limitations in their symbolic or counterfactual thinking skills, following from lack of the necessary linguistic development. As shown above, Norbury suggests that semantic and conceptual knowledge are necessary for metaphor comprehension, and, as such, metaphor use and comprehension is taken to be a complex cognitive task, which, like pretend play, eludes some agents [2]. As poor performance with metaphors is attributed to problems with language, Norbury proposes that “It is not surprising that children with language impairments find metaphor challenging, because they frequently have poor vocabularies and impoverished semantic representations for the words they do have” [2] (pp. 385–386). Difficulties in the comprehension of metaphors is, according to Melogno et al. [28], well documented in high-functioning children with ASD, though they acknowledge that explaining impaired metaphor comprehension in children with ASD is still an open issue. As they clarify,

*“Faced with metaphors, children with ASD tend to remain firmly anchored to literal interpretations. For instance, if one of two children say “Autumn leaves are butterflies,” the listener might interpret the relationship between the two terms (X = autumn leaves, Y = butterflies) literally, i.e., as an identity, and fails to understand the speaker’s intended meaning.”*

*[28] (p. 1)*

Moreover, “difficulties and delay in understanding symbolism, especially in relation to symbolic play, have long been documented as characteristic of people with ASD, and as possibly contributing to social difficulties” [6,29] (p. 145). Jarrold and Conn explain that “among typically developing individuals, language skills in particular are clear correlates of level of pretend play (…), and the same appears to be true in autism” [7,30] (p. 310), though they acknowledge that “there is certainly evidence to suggest that individuals with autism can produce, and make sense of, pretend play in some circumstances” [7,30] (p. 317). 

Connected to the linguistic perspective on imagination of autistics are the types of interventions proposed to train imagination and metaphor understanding of autistics. Typical imagination and metaphor learning programs involve linguistic training. For instance, Gentner [31] asked autistic children to interpret different types of metaphor (attributive, relational or double). As Norbury explains, “Attributive metaphors (‘a cloud is a marshmallow’) were those in which the comparison between two entities was based on shared physical properties. Relational metaphors (‘the moon is a lightbulb’) involved more abstract knowledge about non-physical characteristics, such as function. Double metaphors allowed either interpretation” [2] (p. 385). Happé [32] asked of children to complete six sentences in each of three conditions: synonym, simile, and metaphor. According to Norbury’s analysis, these studies suggested that “as children acquire more abstract semantic knowledge, they are better able to support metaphor comprehension” [2] (p. 385). The focus on linguistic training methods has prevailed since. For instance, Mashal and Kasirer [23] proposed the metaphor training method to include renaming exercises aimed at enhancing semantic flexibility, whereas Pinto et al. [33] devised a Junior Metaphor Comprehension Test, which assesses the ability to explain the meaning of metaphors included in sentences and contextualized in stories. Melogno et al. [28] followed up with an assessment to test the figurative language of children on the autism spectrum, and concluded that “it is possible to teach explicit procedures to enhance metaphor comprehension in children with ASD” (p. 7). To investigate acquisition of prefrontal synthesis in children, Vyshedskiy [5] used the Linguistic Evaluation of PFS (LEPS) test, which “includes flexible syntax and language recursive elements, such as spatial prepositions, to present participants with a set of novel questions that participants have never encountered before” (p. 101). 

For instance, one of the existing interventions for clients on the autism spectrum that targets voluntary imagination is the Mental Imagery Therapy for Autism (MITA) [1,13,26]. MITA is a tablet-based therapeutic application for children with ASD. MITA exercises are designed to “develop a child’s ability to notice and respond to multiple cues presented simultaneously” [26] (p. 3) and train a child’s ability to combine mental objects, and to develop their voluntary imagination. It involves mainly verbal, vocabulary-building exercises, although it does involve some nonverbal, visual exercises as well. MITA aims “to train receptive language, starting with simple vocabulary, and progressing towards higher forms of language, such as adjectives, verbs, pronouns, and syntax” [26] (p. 3); “MITA verbal activities use higher forms of language, such as noun-adjective combinations, spatial prepositions, recursion, and syntax (…) to train voluntary imagination. (…) MITA nonverbal activities aim to provide the same voluntary imagination training visually through implicit instructions” [1] (pp. 7–8). The idea behind how such training works is that lateral prefrontal cortex (LPFC) “can synthesize the objects from memory into a novel mental image according to grammatically imposed rules” [1] (p. 30). Both verbal and nonverbal exercises are hypothesized to be able to improve language ability, and both exercises are aimed to teach the client “to integrate mental objects in novel ways” [1] (p. 4).

In line with the assumption that understanding spatial prepositions requires mental stimulation or mental synthesis (which we can, for all purposes, consider as an imaginative capacity), the idea is that these imaginative capacities are required to develop linguistic skills such as understanding spatial prepositions. Vyshedskiy et al. explain that “understanding of spatial prepositions such as in, on, under, over, beside, in front of, behind requires a subject to synthesize several objects in front of the mind’s eye. For example, the request ‘to put a green box {inside/behind/on top of} the blue box’ requires an initial mental simulation of the scene, only after which is it possible to correctly arrange the physical objects” [1] (pp. 30–31). 

Can we reverse this idea? That is, if we develop such linguistic capacities, will the imaginative skill necessarily follow? Existing interventions seem to assume that the latter relationship holds as well; for example, the verbal MITA exercises that focus on linguistic training are done in the hope of developing voluntary imagination. While it might be the case that imagination exercises directly train neural networks essential for language, there is nothing “linguistic” about imagination itself that does this. From accepting that “exercises training imagination are an indispensable component of language therapy” [1] (p. 43), it does not follow that training language skills allow for the development of imaginative capacities or skills of mental imagery. While that could be the case for some imaginative capacities, such as some forms of suppositional or counterfactual thinking via imagining hypothetical situations or engaging in thought experiments, it is not necessary for all types of imagining. For instance, it is not the only way to enhance metaphoric thinking, as we will show in this paper.

Moreover, voluntary imagination, as characterized by Pearson [13], does not imply that imagination has a linguistic nature. That is, understood as willingly creating a sensory experience, voluntary imagination does not imply that the imagining is tied to language. There is conceptual space to consider what we will term embodied and enactive imagination as a kind of voluntary imagination. In the next sections, we will elaborate on our take on embodied and enactive imagination and metaphors, before returning to the question of how these concepts can inform interventions for children on the autistic spectrum.

## 3. The Embodied and Enactive Turn in Imagination

In this section, we will present an alternative approach to imagination, followed by a discussion of imaginative engagements, such as metaphor use from the perspective of embodied and enactive cognition in Section 4.

Embodied cognition makes use of the concept of body-schematic processes—non-conscious, subpersonal sensory-motor processes that control movement and are involved in habitual know-how and bodily skills [8]. They not only define the possibilities of bodily movement and play a significant role instantiating skills [34], they also influence judgment and decision making [35], and are said to inform even conceptual or complex uses of imagination, such as metaphor use and abstract conceptual thought [36]. On a “weak” conception of embodied cognition, embodied imagination refers to the idea that imaginative processes, including mental imagery understood as representations or models, are grounded in embodied motor activations, understood as neural simulations [37]. On a “strong” or enactive conception of embodied cognition, however, embodied imagination, is not reducible to activating neural representations [8,38]. With the enactive view, imagination is “something that requires active participation, a form of action and interaction” [39] (p. 319). For example, to visually imagine a room, one is “enacting or re-enacting seeing that room, moving our eyes around it and checking things out, and not simply seeing a picture-like mental image of the room with our mind’s eye, or having a mental model of it” [39]. Available proposals for enactive imagination [39,40,41,42] treat imagination as a form of action that is strongly integrated with perceiving, involves embodied sensorimotor processes (constrained by the way the body is, such as the structure of our visual systems [43]), and is densely textured in a cross-modal way, where kinetic/kinesthetic, tactile, nociceptive, even olfactory and gustatory dynamics may be pertinent [38,40]. 

For example, to imagine where to place a crib in the room in anticipation of the arrival of a newborn baby, we do not just imagine the objective space of the room, or the size of the crib or the placement of the windows and the doorway. Rather, we are likely to imagine how we might move around the room if the crib was positioned there, or how we could reach to open the window with the crib placed on that wall [38]. In this regard, our imaginings are affordance-based. In the imagining process, our body and the way our body moves play a role: the imaginary movement and our imagining of the room involve an activation of some of the same sensory-motor processes that would be contingently involved in, and would constrain, our actual movement if we were actually moving.

In this respect, a person in a wheelchair, or one who operates with a different set of sensory-motor contingencies, might imagine the room arrangement somewhat differently. A person on the autism spectrum might also have their own, unique way of imagining, due to their own unique set of sensorimotor contingencies. This proposal is in line with growing evidence that ASD involves significant differences in sensory-motor processing related to motor control (specifically disrupted patterns in afferent and proprioceptive sensory feedback [44,45,46]), postural instabilities, atypical gait, mistiming of motor sequences, motor coordination problems, and problems with anticipatory postural adjustments [47,48,49,50,51,52,53,54], all of which may have implications for embodied imaginative capability. We can view this as a difference in, rather than an impairment of, voluntary imagination. Just as there may be some differences in the perception and imaginative processes between subjects due to different patterns of sensory-motor contingencies related, for example, to different skill levels, so there may be differences between people on the autism spectrum and those who are not on the spectrum. 

Importantly, imaginings are always, for both weak and strong embodied cognition, shaped by sensorimotor processes. What I imagine is shaped by my bodily possibilities (what sorts of movements I can make and the kinesthetic sensations that may accompany those movements) and perhaps by specific past bodily actions (hiding, climbing, running) that may have honed my skills. For instance, even imagining “putting a green box {inside/behind/on top of} the blue box” (a case described as a combining of mental objects) may involve sensorimotor processes rooted in past bodily actions of placing objects inside or on top of another, or one’s own bodily experiences of hiding behind other objects. 

The findings from the neuroscience of motor imagery further support the argument for the embodied account of imagination, as they also characterize mental imagery as a motoric, bodily process, involving neuronal activation in motor areas. While neuroscientists may use the terms “motor imagery” and “motor image”, in the end, what they explain are the various processes and parameters involved in imagining a bodily performance, including perspective, duration of the task, or the length of the performance [55,56,57]. Motor imagery and actual movement share common neural substrates, as motor imagery activates the same neural networks used in perception and motor control, e.g., motor-related ventral and dorsal parts of premotor cortex, supplementary motor area; parietal areas; subcortical areas, including cerebellum and basal ganglia [58,59,60,61]. Motor imagery can involve the visualization of an action, or the imagining of how a movement feels; it can be done from a first-person or a third-person perspective, and it can improve motor performance and enhance motor recovery from injury by activating neural substrates of movement and producing minimal movements, such as muscle contraction (for more on the relationship between motor imagery, neural activations and motor performance, see [62]). 

Here, we can reference the evidence not only for the fact that motor imagery is rooted in motor processes, but also for the fact that experiences gained from explicit motor performances can enhance motor imagery. Most clear evidence for the role of explicit movement for the capacity to engage in new imaginings stem from sport studies [63] and performance studies [64]. Thus, calling imagination “embodied” not only intends to capture the fact that imaginings involve neuronal activation (that would be trivial), or that they somehow refer to the historical role of the body [65]. Rather, there is a much more explicit role for the body in shaping imagination, in that explicit and occurrent movement can further infuse our imaginings and bring forth new imaginative experiences. We can call this explicitly embodied imagination. Explicitly embodied imagination involves the body shaping and maintaining imaginative processes in a way that cannot be uncoupled from the ongoing interactions with the environment. Simply put, movement further allows us to engage in new forms of imagining as it allows us to imagine situations that were unimaginable before—for instance, imagining how a mathematical equation adds up, or how the planets or meteors move through space [66]. We can find examples of explicitly embodied imagination in situations of symbolic and pretend play, and in performance practices of marking (in dance) and blocking (in stage performance) [38]. In these contexts, imaginings are actively shaped by the use of artefacts, props, and engagements with other people, leading to jointly discovering and creating new possibilities for action. This leads us to alternative understandings of the relationship between imagination and metaphor use.

## 4. Enactive Metaphors

The linguistic approach to metaphors (as discussed in Section 2) is not the only available approach to understanding metaphor comprehension and production. The alternative view is that metaphor creation also rests on embodied, perceptual and enactive capacities. That metaphors can build on embodied experiences has been famously proposed by Lakoff and Johnson [36,67], who clarified that the language that is developed in metaphor production is grounded in physical experiences. Although their conception of metaphors is not in itself enactive, their main concern was to establish the bodily basis of seemingly abstract or disembodied concepts, an idea that is built on by the enactive approaches to metaphor as well. They propose that human beings “inevitably acquire an enormous range of primary metaphors just by going about the world constantly moving and perceiving” [67] (p. 57), demonstrating, thereby, that there is a strong role of embodiment in the formation of metaphors that lead to abstract concepts. Some examples include experiencing the body as a “container”, life as a “path”, or things we need to look up to as “good”.

Other approaches to metaphor production and comprehension, that move further away from the linguistic approach, are embodied approaches, such as that of Littlemore [68], which focuses on metaphors as triggering a strong physical association, eliciting neurological responses and incorporating sensory experiences, or Neisser [9], who proposes that “metaphor is an activity for coping with the world. It is how we make sense of things. Even when subconscious, metaphor is an interpretive strategy—a heuristic device which opens up new symbolic and cultural fields” (p. 38). There are also novel ecological approaches to metaphor that stress the perceptual processes at the heart of metaphors, explaining that these involve direct pick-up of an invariant pattern in an informational array specific to both the source of the metaphor (e.g., fireworks) and its target (e.g., “flowers on the sky”), thereby not relying on complicated cognitive tasks to make the connection [69]. 

Furthering these embodied approaches to metaphor, we will focus now on the enactive approach which proposes a different way to think of metaphoric engagements. Instead of passively encountering metaphors that require the agent to think through the mappings from source to target domain, Gallagher and Lindgren [66] propose that we can enact metaphors, or bring them into existence through action. For example, they describe the experiment "Meteor", where the agents (school age children) entered a virtual reality simulation and engaged with a virtual meteor, metaphorically identifying with it (“I am the meteor”) by acting to control the meteor to learn about its movements and predict its trajectory. The children embodied the meteor, running and jumping to control it through explicit navigation of the space around them. In the context of the game, the children’s running had a metaphoric nature, as the running enacted the movement of the meteor. Gallagher and Lindgren [66] found that physically enacting the meteor’s movement led the agents to a more comprehensive and flexible understanding of some critical ideas and principles from physics about movement through space (such as gravitational acceleration and Kepler’s Laws of planetary motion). This provided new insights about the benefits of full-bodied enactive participation for learning environments. In short, the enactive approach to metaphors saw metaphoric engagements as forms of pretend play or acting-as-if one thing is another. It involved a metaphoric transformation of the target into a source. Importantly, the metaphor at stake was not “sitting someplace in her head; it’s [rather] in the movement that [the agent] makes (…). She constitutes the metaphor by her action” (p. 396). This is in line with Neisser’s [9] analysis that “By themselves metaphors don’t work very well (…) Metaphors, rather, can provide models of gestalt structures—feels and styles—rather than rigorous definitions and rules. Metaphor is most useful as a way to induce sudden insight, like a flare that momentarily illuminates the whole territory around, not just the road ahead” (pp. 38–39). 

Following this enactive approach to metaphors, we propose to see metaphors not as linguistic tools, but as affordances for actions. Seeing metaphors as affordances allows us to use metaphors (existing or created) to bring about new bodily experiences. Metaphors can be used to create new kinds of visualizations, and feelings that come with it. They offer possibilities for new ways of engaging and give us the opportunity to approach and experience the target in a different way. This is the case not only for explicitly embodied performances, as seen with the Meteor case, but can refer to the act of speech itself: talking metaphorically is a way to enact metaphors.

In our view, the meaning of a metaphor is flexible as it takes shape within the context in which it is used and through the interactions from which it emerges. Metaphoric meanings are often not discovered in solitude by conceptual analysis, but are co-construed in play with others, as well as in dialogical interactions, through participatory sense making processes. That is why metaphors can be “reused” but can take on a different meaning in different contexts. Metaphoric engagements understood this way are enabled by imaginative capacities that are embodied in the sense discussed above. We engage with metaphors not just thanks to detached linguistic analyses and “offline” simulations of mental contents, but thanks to the triggering of sensorimotor responses at the roots of metaphoric engagements [36,67], as well as in overt behavior, in, for example, instances of pretend play [8,10,66].

In the next section, we will show examples of enacting metaphors in therapeutic engagements with children on the autistic spectrum. In the final section, we will conclude with suggestions for further interventions, based on the idea that imagination is embodied.

## 5. Metaphoric Engagement with Children with ASD in Systemic Therapeutic Conversation

In this section we will provide examples from a practical therapeutic session with an autistic child that makes use of metaphors. We will provide extracts and an analysis of a therapeutic conversation between a twelve-year-old boy, Noah, and his “systemically-oriented” psychotherapist. Through the extracts of the conversation between Noah and his therapist, we want to show how Noah, despite his diagnosis of autism, spontaneously uses several metaphors in order to share his experiences with the therapist, and how the therapist builds on those metaphors to enhance communication between them. This example does not aim to provide empirical support to our theoretical insights and should not be confused with a case study. We intend for this example to serve as a mere illustration of how enacting metaphors works in practice, and what further insights it can provide about metaphoric engagements. While limited in its scope, we think that it shows that it is important not to generalize the idea that people with autism have difficulties with metaphor use (or imaginative engagements), and that approaches to metaphor use which apply the enactive approach to metaphors and imagination, such as those found in systemic therapy, can offer new ways of helping people with autism.

We begin with a short explanation of “systemic (psycho)therapy”. Systemic therapy is a process-oriented way of working, in which the focus is on creating changes in how a person experiences himself in relation to others. The therapy aims to create a context in which the client and the therapist can reflect together on the effects of the numerous interactions in which a client is interwoven outside the therapy room [70,71]. The focus of the therapy is thereby not on enhancing individual (cognitive) skills of the client, such as acquiring knowledge, changing thought patterns, or even teaching metaphoric thinking. Its aim is not to teach the autistic person new skills, but to jointly reflect on and take new perspectives on the clients’ encounters. Its focus is on jointly creating a space where the client and the therapist experiment with “new ways of positioning themselves”, which means trying to find novel perspectives on the problems the client faces so that new action possibilities for the client can emerge. The systemic therapist can choose to use metaphors to be able to talk with his/her clients, targeting not the development of the metaphoric repertoire of the client, but the client’s own feelings and experiences. Using metaphors in dialogue and play for this purpose allows the client to reflect with the therapist on encountered difficult situations, as will be explained below. Dialogues and play in systemic therapy allow for the discovery of new meanings, in order to facilitate the development of novel perspectives [72]. This stance is consistent with the enactivist insights that words do not have static meanings, and that the meanings of actions (such as play) are not “hidden behind those actions”; instead, the meanings of words and actions “emerge out of” interpersonal discussions and interactions [72] (p. 2). Systemic therapists also refrain from attributing prescribed meanings to their clients’ behaviors. They work with a “not knowing stance” [73] and try to refrain from analyzing the words and behaviors of their clients; instead, they pay attention to their descriptions and introduce, in dialogue and in play, new ways of talking in order to change the way their clients relate to others and to the struggles they face. 

In general, systemic therapy sees people as nodes in relationships. It does not situate the problems people are struggling with “inside their heads”, but rather, in the unique interactions between people and the numerous contexts in which they live. Well-being, from a systemic perspective, arises when people feel understood and valued by others, in and through the many interactions they engage with, and when they can maintain a sense of agency or influence over their lives [74]. The sense of agency is an embodied “feeling” of being able to act, to do, to move, to change. The systemic therapist aims to give its clients the feeling of having a grip on what they are struggling with, not only during their interaction, but extending beyond the therapeutic room and into their lives. The systemic framework, therefore, fits seamlessly with the embodied and enactive take on cognition, which also emphasizes that cognitive capacities are not reducible to brain processes, but that cognition should be situated in dynamical processes in which brain, body, and the social and physical environment are continuously connected with each other [8]. It is important to note that systemic therapy is not an intervention specifically aimed at people diagnosed with an autism spectrum disorder. However, it can successfully contribute to the development of the sense of well-being of autistics as well [75], as will be shown below. 

From a systemic perspective, metaphors offer a significant added value to the psychotherapeutic process. The use of metaphors in psychotherapeutic contexts is neither new nor intrinsic to systemic therapy [76], and there is no systemic view of metaphors as such. However, systemic therapy can rely on the enactive view of metaphors to guide its practice. That is because they share the common assumptions about what it is to think and to imagine: they both accept that thinking and imagining are processes that occur in acting. This will also hold for symbolic thinking and metaphor use.

A systemic approach inspired by an embodied and enactive account of metaphors emphasizes how metaphoric engagement can be seen as a context (a “linguistic world”) within which the therapist and the client stay in order to explore their experiences and create new ways of positioning. This context is a jointly created space where the exploration of the meanings and the change in perspectives can occur. From this point of view, the therapist pays attention to the metaphors that the clients spontaneously use when talking about their experiences. The therapist also asks clients for metaphors that suit their lived experiences. Subsequently, the clients are invited to further engage with the metaphors produced in the interaction with the therapist, by questioning and reflecting on them. The therapist also gains an alternative way of listening to what clients have to say and how to respond. To facilitate the process of engagement, the client and the therapist often try to find ways of externalizing the metaphor, by drawing it or using figurines and objects to stand in for the metaphor.

A systemic approach also implements, in practice, the idea that metaphors themselves are affordances for actions, emerging in relational contexts. From the systemic perspective, metaphors function as therapeutic tools that ease communication and that the therapist and the client can elaborate on together. Metaphor, understood as a tool for engagement, follows the enactive idea that sees metaphor as a “product of an organism-environment-system—rather than merely a product of an inner mental process” [77]. We think that metaphoric engagements in systemic therapy only work when they are part of a collaboration. Making an analogy through the metaphor is in itself not sufficient for therapeutic gain, because it is not the metaphor itself (its symbolic properties or the fact that it builds on a perceptual resemblance between the source and target domains) that does the work; it is how the metaphor is used and responded to in an interaction that does the work. The work in question involves discovering new perspectives and repositioning oneself in relation to one’s life struggles. What matters, then, is how the metaphor is received and what is being done with it. This is in line with Gallagher and Lindgren’s [66] idea that “metaphors do things, but only when we engage with them in some fashion”. 

We will now provide an example from the therapeutic practice with an autistic child, Noah, in which his spontaneous use of metaphors, and the therapist’s capacity to pick up on those metaphors and continue the conversation with them, was important for the success of their therapeutic engagement. 

Noah was diagnosed with autism at the age of eight. His parents contacted the systemic therapist to help Noah work through his doubts, worries, and anxieties through conversation, thereby hoping to mitigate Noah’s low self-esteem, sadness and anger outbursts. In the therapy room, at the start of one of their conversations, Noah tells the therapist about a big argument he had with his mother. Noah explains how, during that fight, he used words that hurt her. The therapist decides to help Noah visualize the conflict by taking out two small figurines to represent Noah and his mother, and putting them on the table in front of him. The therapist repeats what Noah said while pointing at the figurines and adds that he recognizes that people sometimes say hurtful things to each other in a quarrel, while at the same time still continue to love that person. Noah answers:

Noah: *Yes, on the outside, people remain the same, but on the inside, people “switch” to another position when they are angry. … Look, I’m standing here and she’s standing there. And she gets angry with me. She’s comes too close and I want to push her away.*

Noah then moves the figurines to show what he means. He takes the figurine of the mother and moves it in the direction of the figurine representing the boy. Then he takes the figurine of the boy and uses it push away the mother. Noah continues.

Noah: *When I “switched” to that other position, I still wanted her to be my friend, but at the same time I wanted to push her away. And then she builds a kind of a “wall” around her. Do you know what I mean?*

Notice how Noah spontaneously thinks of and uses two different metaphors in this one interaction: the metaphor of the “switch”, which stands for Noah’s shifting between attitudes, and the metaphor of building a “wall”, which stands for the psychological difficulty Noah experienced in communicating with his mother. The metaphors seem to help Noah to express what he believed happened and to give words to his experiences. 

The therapist then decides to ask Noah a question to further explore Noah’s experiences. He does so, however, by staying within the context of the metaphor.

Noah: *When I’m angry, I want to push someone away. I don’t know why, but it’s just out of anger. And then afterwards it turns out to be worse than before.*

Therapist: *I understand. Now, when your mum stood over there at a distance, what happened to your “switch”? Now that I come to think of it, how many “switch” positions do you think people have?*

Noah: *I think people have three positions: normal, angry and relaxed.*

Therapist: *Okay, so when your mum stood back there, did your “switch” stay on ‘angry’, or did it switch to another position?*

In continuing the conversation, the therapist plays on the fact that switches can have different sizes, colors, number of positions and ways of handling them. The aim of the therapist is to stay within the context of the metaphor, as this context opens up a linguistic world of possibilities for discussion and allows both of them to explore Noah’s experiences in novel ways.

Finally, while exploring the idea of “switching” moods, Noah sees a resemblance between what happened at home and what sometimes happens at school. At school, he also experienced people coming too close to him, and experienced his “switch” turn to the angry mode, resulting in him pushing those people away. He noticed that, like his mum, his peers also pull up a “wall” around them. 

Triggered by the fact that Noah uses the metaphor of the “wall” again, the therapist stands up and takes out a box with colored cubes. He takes out three cubes, coincidentally all three having a different color, and stacks them between the two figurines. His intention was to use the blocks to facilitate a more in-depth exploration of the metaphor of the “wall”. He adds:

Therapist: *So that’s your wall. But maybe you want to build it? How high was that wall?*

The therapist stays within with metaphor but uses action-verbs such as “to build” to further engage Noah in thinking about his problem.

Noah, however, grabs the three blocks, straightens them and puts them next to each other in front of the figurine that represents him. He says:

Noah: *Look, that figurine is me, and the red block is ‘angry’, the blue block is ‘normal’ and the yellow block is ‘relaxed’.*

Although the therapist intended to use the cubes to represent the wall, Noah did not pick up on that suggestion at first. Instead, he uses the cubes to represent the three modes of the switches to further discuss what happened between him and his mother. The therapist and Noah continue to talk in metaphorical terms, but for a moment, they give different meanings to the objects. 

However, they soon realign, as Noah spontaneously changes the meaning he originally assigned to the blocks (from switches to wall bricks), and stacks them to represent a wall between him and his mother. From that point on he talks about a “wall” between him and his mother, although he keeps the distinction between ‘normal’, ‘angry’, and ‘relaxed’ modes from the previous metaphor. He describes that when he is feeling normal or relaxed (which he shows by dropping the block down), his mother comes closer to him (which he shows by moving the mother figurine closer to his figurine), and when he is angry, his mother keeps her distance and builds a wall between them. Noah then discusses with the therapist how his mother’s building a wall between them affected him, and explains that the harder he pushes to break the wall, the firmer the wall stands between them. However, the moment he withdraws for a while, he notices that his mother slowly takes the wall bricks away, one by one. Noah realizes that this is when he and his mother are able to talk to each other again. 

We will now provide some interpretations of the abovementioned metaphoric interaction from the embodied and enactive perspective on metaphors, and suggest new insights that follow from taking this perspective.

The fact that Noah spontaneously uses metaphors such as “switch” and “wall” shows that metaphors are grounded in embodied experiences. From an embodied perspective to metaphors, we can make sense of why Noah uses these metaphors: they correlate with his lived experiences. Noah undoubtedly has experiences with switches and knows how to switch something on or off (as in a light switch), or how to switch a device to a higher setting to increase the intensity with which a device works (such as the air conditioner). Noah also has experience with the solidity of walls and understands that walls separate places (and sometimes people). These concrete experiences afford Noah the possibility of using them in a metaphorical way in order to be able to express his experiences to the therapist. This resonates with the idea that metaphors in conversations work not necessarily due to the perceptual resemblance between the source and target domains, but because they relate to embodied experiences of people. As Mark Johnson [78] claims,

*“contrary to the reigning comparison theory, metaphors are not typically based on perceived literal similarities between to different domains, but rather are based on experienced correlations between the source and target domains (p. 15).”*

Johnson [78] further clarifies that the individual need not necessarily physically live those experiences. It suffices that the experiences are “imaginable”:

*“Any aspect or quality of a situation (…) is called forth by way of experience. That includes past experiences, present experiences, and projected future experiences perceived to be possibilities developing out of one’s current situation (p. 19).”*

The embodied-enactive view of imagination can make further sense of this idea—that even the imaginative projections of future possibilities for actions are grounded in past sensorimotor experiences [62]. To perceive affordances for action, or possibilities that can develop out of one’s situation, is an embodied and imaginative activity, as perception of affordances is an act of anticipating what actions can be afforded based on past sensorimotor experiences. Thus, the metaphoric engagements described above rest on the capacity of the interactants to imagine possibilities for action, as rooted in and shaped by their past sensorimotor experiences. 

The metaphoric meanings of the objects also changed with active movement and play. The explicit movements in the play (from stacking up the blocks vertically, to dividing them up horizontally) corresponded with the metaphor that was used at a time, and influenced how the metaphoric narrative developed in the interaction. Explicit movement allowed the pair to imagine a new situation, in line with the idea of explicit embodied imaginings discussed in Section 3.

Moreover, we see that the use of metaphors in this therapeutic encounter was not directed only at “uncovering the experience” of the client, but at changing those experiences. The therapist tried to capture the sense of what is being said through the client’s metaphor, but also to use that same metaphor to think about possible future states. For example, the “switch” metaphor offered a visualization of the issue of anger and talking about ways of acting on it. After Noah compared his anger to switches, the therapist could use this idea to get to know Noah’s emotional state better, by asking questions, such as “how many switch positions do people have?” or “what happened to your switch when your mom was standing there?” It also allowed the therapist to play with the received metaphor, from the idea that one can switch between attitudes (angry—not angry) to the idea that there are different positions on a switch, as in a light switch. Such engagement can be seen as a needed intervention, as it allows for stepping outside of the “entrenched” stories and experiences. The metaphoric engagements thus help us escape static ways of conceptualizing our problems, which can create a sense of powerlessness. Metaphors offer a way to get “unstuck”, especially when they are used in a pair with action-verbs. 

Importantly, it is not just the metaphor itself, such as using the word “switch” to stand in for anger, which helps to achieve the therapeutic gain; “the switch” on its own does not suggest anything about a bodily positioning towards anger which one can take. It is the therapist with his questions about how to act on these objects that helps to make the metaphor action oriented. While some metaphors are verbs (“to switch”), often metaphors come in the form of nouns (“wall”). Other examples of metaphors-as-nouns found in this systemic therapist’s interactions include “tattoo” to stand for auto-mutilation, “shield” to stand for protection, or “domino blocks” to stand for inevitable chain of events. Even then, the systemic-enactive approach proposes to focus on actions associated with these nouns. For example, the “switch” metaphor can suggest the action of switching or changing one thing to another, whereas “the wall” metaphor can suggest the action of building, climbing over, breaking or looking beyond it. As verbs presuppose action capabilities, all metaphors in systemic therapy should be treated with the additional use of verbs. 

Using metaphors in such a way offers clients the possibility to imagine being able to change or handle these situations. These newly created imaginings, in turn, are not purely hypothetical, but enacted, in the sense that they can trigger action responses, because they are connected to action capabilities. Consider the emergent metaphor of the “wall”, which represented the problematic relationship between Noah and his mother. By realizing that he and his mother are able to talk to each other again as he waits for her to take the "wall" down slowly, brick by brick, instead of him trying to push the wall down, Noah gains new insights on how to communicate with his mother. The intended result of metaphoric engagements is to allow clients to regain a (renewed) sense of agency, or a sense of influence over their lives and over the struggles they face, even if they cannot fully control them. In the case of Noah, realizing that the problem lies in his style of interaction with his mother, and not just with him, helped him raise his self-esteem.

Thus, from the above descriptions, we see that we should not think of metaphors as static linguistic or symbolic thoughts simply “sitting in the head” of one individual. Rather, we need to think of them as tools in a metaphoric interaction, intersubjectively developing between individuals. Metaphoric interactions are joint processes of exploration and sense-making—a process of subjective understanding of the world. De Jaegher and Di Paolo [79] have termed this “participatory sense making”, which refers to the idea that our social interactions change how we perceive and understand the world. No individual person chooses their own meanings of experiences; experiences become meaningful in interaction with others, negotiated within a cultural practice. As part of sense-making engagements, metaphors are not just symbolic pictures, but should be seen as objects and environments that can be acted on and shared. 

Finally, the example with the blocks acting as metaphors for the “switches” at one time and the “wall” at another time shows that metaphoric meanings are negotiated in action. It is likely that at first Noah was triggered by the fact that there were exactly three blocks (as many as the positions of the switch he mentioned earlier), each with a different color, that impacted his decision to continue to talk about them as “switches” and not as “walls”. The meaning of the block was flexible and took shape within the context of the interaction. Talking metaphorically challenges the therapist to coordinate this joint process of exploration and meaning making in order to ensure that with their client they are still talking about the same topic. Firstly, therapists must ensure that they and their clients are both still talking metaphorically. Secondly, they must ensure that, while talking metaphorically, they are still talking about the same thing. For example, while they are talking about “switches”, they must both be aware that they are in fact talking about the client’s feelings. Sometimes this coordination process fails and requires both parties to realign, just as it happened in the conversation between Noah and his therapist.

What insights about conversational capacities of autistic people follow this example? It is possible that the coordination and communication processes between autistic and neurotypical people sometimes fail due to the reduced intersubjective engagement and responsiveness of people with autism, along with a possible impairment in joint attention and other forms of “secondary intersubjectivity” [80,81]. Typically, interacting subjects attune to each other and are sensitive in re-aligning themselves in a conversation when they sense that they are getting off track. The enactive approach to social cognition, which focuses on embodied interactions, proposes that in our ordinary everyday interactions, people make sense of each other without theorizing or simulating each others’ mental states; they recognize what the other person intends or feels in the other’s bodily postures, movements, gestures, or intonations [51]. As Gallagher and Varga [50] write,

*“Processes of primary intersubjectivity that involve perception of the other’s bodily postures, movements, gestures, facial expressions, vocal intonations, etc., as well as dynamic embodied interactions, often in rich pragmatic and social contexts, allow young infants, as well as adults, to gain a basic sense of the intentions and emotions of others (p. 4).”*

Hence, we are not required to engage in implicit processes of mindreading, simulating or theorizing about the others’ mental states; Theory of Mind capacities need not be invoked. Intersubjective engagements are often sufficient for having a sense that we are talking about the same thing when using a metaphor. While “knowing you’re talking about the same thing” and “having a basic sense of the intentions and emotions of others” might be considered to be different capacities, enactive theory proposes that it is thanks to alignment and through continued coordination that we would soon discover linguistic misunderstandings, such as by seeing that the person we are conversing with looks puzzled.

When it comes to people on the autism spectrum, it is likely that their ability to follow each other without explicit signposting of conversational dynamics is lower than that of neurotypicals. For example, in the case discussed above, we can see that after Noah introduced the “wall” metaphor, he explicitly asks the therapist “Do you know what I mean?”. This suggests that, on the one hand, Noah was paying attention to whether he is understood by his conversation partner (showing that he is an active member of the conversational dynamics), but on the other hand, he explicitly sought a verbal confirmation of that understanding. It might be that the therapists’ nodding or other non-verbal responses were not sufficient to keep the metaphoric engagement going, which is in line with the finding that children with ASD show less engagement with the bodily-expressed attitudes of others [82].

Thus, it may be that, sometimes, the difference between the systemic therapist engaging metaphorically with the autistic child vs. with a neurotypical child is that the interaction with the former requires more attention from the therapist on whether the shared ground is kept. This is in line with Iverson and Wozniack’s [83] insight that targeted interventions with autistic individuals should focus on enhancing not their individual cognitive capacities, but their communicative environment, which can be achieved by encouraging the caregivers (and therapists) to “respond consistently and contingently to communicators’ gestures and nonword vocalizations” (p. 66), and in general, to pay special attention to the non-verbal aspects of the conversational dynamics in which they jointly engage with their autistic clients.

However, importantly, it is not the case that metaphoric engagement must be off limits to children with ASD to begin with. We will conclude with insights on the topic of imagination of autistic children below.

## 6. Conclusions

We conclude by summarizing some insights about metaphoric and imaginative skills of people with ASD that follow from taking the embodied-enactive perspective on imagination, followed by suggestions for interventions. 

To summarize, we have proposed that imagining is grounded in embodied motor activations (not just cognitive, semantic operations): it is grounded in how we perceive the world through our senses and in how we move around and experience the world. We have shown that the capacity for combining mental objects or creating new combinations “in mind”, as seen in the example of metaphoric engagements, can be usefully interpreted as an embodied capacity. We can make sense of it in the notion of enactive metaphors. Seeing imagination fundamentally as an embodied and sensorimotor activity can make sense of how we engage with and integrate mental objects in new ways. Embodied imagining is still voluntary, as our movements and actions in response to affordances actively shape our imaginings. Imaginative experiences are never dependent on the person’s cognitive mechanisms alone but are situation dependent and co-created in the interaction. 

In addition, we proposed that metaphoric engagement is an act of sense making in which the subject makes use of an experienced correlation between source domain and target domain. These experienced correlations are grounded in embodied activities. Metaphoric engagements are situated engagements and not just language dependent. The key difference in our account of metaphors, in contrast to others, is that we conceive of metaphors as affordances for actions: they help us imagine new experiences and perceptions in the possibilities for action they open up. Metaphoric engagements do not rest on imagining counterfactual situations achieved through complex linguistic manipulations, but on embodied, sensorimotor imaginings. Understanding metaphors does not rest on linguistic or semantic conceptual structures alone, but requires movement-related, body-schematic structures, and interactive contexts, which, in turn, may shape the linguistic capacities associated with metaphoric thought. As Neisser [9] proposes,

*“It is the qualitative value of the commonplace associations and implied inferences which give a metaphor its force—its value for us. Its significance lies not so much in its sheer conceptual structure as in its relation to an embodied agent, and the way it enters experience through imagination. (…) [The] key to this “taking up” of experiential qualities into linguistic cognition lies in the body scheme, which plays a synthetic or productive role in imaginative thought (p. 36).”*

Thus, while from the perspective of the linguistic framework, metaphor use and comprehension of people with ASD should be severely limited before they gain linguistic training, the embodied/enactive view clarifies why this need not be the case, as conversational dynamics of systemic therapy show. From our perspective, therapeutic interventions that make use of metaphors and symbolic play are possible forms of engagements with clients on the autism spectrum. This can be explained though the importance of creating a context that affords imaginative engagement in the form of metaphoric engagement. 

Consider again Vydshedskiy’s [5] proposal that voluntary imagination is the mental juxtaposing of objects into a novel image, that there is a strong connection between voluntary imagination and language comprehension, and that by training language comprehension, imagination can be improved. Although these claims may be true to some degree, we propose to situate imagination in a broader field of interactions and influences. From an embodied and enactive perspective, imagination is grounded in linguistic interactions with others, in sensorimotor engagements, and imaginative engagement is dependent on what a situation affords in terms of possibilities for action. 

It follows that, for instance, the distinction between voluntary and involuntary imagination is not so straight forward on our embodied-enactive model. When Noah is using metaphors deliberately in a conversation, this suggests that the imaginative act is voluntary. At the same time, the metaphor he uses need not be deliberately planned or thought through in advance of the conversation, it emerges in response to the dynamic interaction with the therapist, and often is based on embodied schemas that are at the root of the association that can be drawn between the metaphor and its target, which suggests lack of voluntary (or fully controlled) processes. The embodied and enactive perspective shows that drawing a clear-cut distinction between voluntary and involuntary imaginative processes is difficult and perhaps counterproductive. Instead, we should think of those processes as dynamically unfolding over time, in action and interaction. Imagination may involve both neurological "top down" and "bottom up" subpersonal cognitive mechanisms, but that does not suggest that the imagination itself should be reduced to those terms; it rather should be seen in a hybrid and holistic way. Rather than situating imaginative skills in the brain, or focusing on the brain-bound processes alone, we should think of imagination as connected to action, and metaphoric engagements as meaningful in a particular interactive context. 

This makes a difference to how we could potentially develop new intervention strategies. By situating problems with imagination inside the “brains“, the interactional and ecologically situated nature of imagination is lost. Consequently, intervention strategies become focused on the contextless training of imaginative capacities, rather than on creating contexts in which imagination can be developed and used within uniquely situated interactions. As an alternative explanation to brain-bound semantic capacities, we consider how sensorimotor problems in people with ASD can constrain their capacities for developing and using metaphors. As shown in the previous sections, sensorimotor problems might affect how people with ASD communicate with their therapist, such as whether they attune to their interlocutor, making a difference on whether or not the metaphoric engagement is shared. Nonetheless, people with autism can show imaginative skills; they can engage metaphorically, when in the right dialogical context. Thus, they might be able to benefit from interventions that rest on exercising imagination in action, such as metaphoric engagements and pretend play. 

While we are not in the position to recommend specific interventions or make clinical recommendations, we hope to have inspired future work that does not start with the assumption that people with ASD have impairments of imagination but takes as a starting point the idea that imaginative engagements are, for everyone, context-dependent and action-bound. This suggests that autistic people do not always have to be first trained in imagining counterfactual situations through linguistic training methods, in order to enhance their imaginative skills, and that, perhaps, language-dependent tasks are not always the only or the best options for children with autism to acquire new imaginative experiences. We do not reject the usefulness of the available metaphor training programs. Although children with autism can benefit from training such as MITA, an embodied, enactive and situated approach to imagination can broaden the scope of how we can further support the development of children with autism. If imagination is not so language-dependent, but rather, is dependent on how much a situation affords children to engage imaginatively, then the question becomes: how can we create a context in which a child feels invited to engage imaginatively? Jacqueline Nadel [84] developed training in which she presented children with autism with many different toys, always two of a kind. At first, the trainer who joined the child in the room imitated whatever the child was doing with the second of the pair. Gradually, children with autism noticed this pattern and began challenging the trainers to imitate their play. Once the relationship between the child and the therapist was installed, the trainer added new possibilities to the game by inviting the child to imitate him or her in return, gradually exploring new possibilities in playing with the toys by using them or combining them in different ways. In this case it was not the therapist or trainer imposing the task on the child, but first, there was the child engaging in play that was meaningful to him, after which the therapist connected to the play and added elements to it in order to create new possibilities. 

Accordingly, the enactive approach to metaphor production, building on embodied imaginings and explicit performances without assuming complex linguistic skills on the part of the agent, suggests that it is important for researchers and therapists interested in offering children with autism opportunities to engage imaginatively to acknowledge that imaginings are contextual and rooted in sensorimotor experiences. A focus on conversational dynamics is important for stimulating imaginative interactions, as acknowledged by Vyshedskiy [5]. Our work should thus be seen as a welcome addition to the available perspectives on metaphoric interactions of people with autism spectrum disorder. We invite future studies including clinical and empirical analyses to consider expanding their interventionist repertoire, and augment linguistic treatments that focus on “training the agent to analyze semantic similarities and differences between meanings” [28], or are focused on vocabulary-building exercises (such as MITA), with dialogical engagements and pretend play where metaphors and imaginings can be enacted.

## Data Availability

The data presented in this study are available on request from the corresponding author. The data are not publicly available due to privacy or ethical problem.
